# Policy liberalism and source of news predict pandemic-related health behaviors and trust in the scientific community

**DOI:** 10.1371/journal.pone.0252670

**Published:** 2021-06-17

**Authors:** Madeleine Reinhardt, Matthew B. Findley, Renee A. Countryman

**Affiliations:** Austin College, Sherman, Texas, United States of America; Neijiang Normal University, CHINA

## Abstract

In March of 2020, the United States was confronted with a major public health crisis caused by the coronavirus disease (COVID-19). This study aimed to identify what factors influence adherence to recently implemented public health measures such as mask-wearing and social distancing, trust of scientific organizations like the Centers for Disease Control and Prevention (CDC) and the World Health Organization (WHO) on information pertaining to the pandemic, and level of perceived risk. Data were collected from June 30, 2020 to July 22, 2020 on 951 adult residents of the United States using an online survey through Microsoft Forms. Multiple linear regression was used to identify the strongest predictors for compliance to pandemic-related health measures, trust in the scientific community, and perceived risk. Results showed that the strongest predictor of all variables of interest was degree of policy liberalism. Additionally, participants who consumed more conservative news media conformed less to the pandemic health guidelines and had less trust in the scientific community. Degree of policy liberalism was found to have a significant moderating effect on the relationship between gender and conformity to pandemic-related health behaviors. These findings have concerning implications that factors like degree of policy liberalism and source of news are more influential in predicting adherence to life-saving health measures than established risk factors like pre-existing health conditions.

## Introduction

In March of 2020, coronavirus disease (COVID-19) was declared a pandemic by the World Health Organization (WHO) [1]. The outbreak of this virus originated in Wuhan, China where it quickly spread to the rest of the world. Although this health disaster occurred on a global scale, the United States contains the majority of the world’s COVID-19 cases [2]. This novel virus is expected to become one of the leading causes of death in the United States in 2020 and has caused significant impacts on the economic welfare and physical wellbeing of Americans.

At the time that this research was conducted, no pharmaceutical interventions were available for COVID-19. Presently, *Veklury* (remdesivir) has been identified as an effective antiviral medication for the treatment of COVID-19 and was approved for emergency use by the FDA [3, 4]. As of February 2021, three mRNA vaccines have been developed and distributed to many high-risk individuals with the purpose of further reducing COVID-19 transmission and illness severity. Recent literature on health behaviors related to COVID-19 finds that measures such as social distancing, mask-wearing, frequent handwashing, and use of alcohol-based sanitizers are effective in reducing transmission of COVID-19 [5–7]. Despite concerns over violation of individual autonomy, most states are requiring adults to wear masks in public spaces and indoor areas such as stores and public transit [8–10]. In response to the rise in cases of COVID-19, several states have implemented public health interventions such as limitations on social gatherings and requirements on mask wearing, ranging from loose restrictions to very aggressive efforts to ensure public safety [11, 12].

Enforcing mask wearing is not the only challenge related to reducing the spread of infection. Individuals have received evolving information about the virus as new discoveries emerge. In March of 2020, the CDC did not include mask-wearing as a crucial method of preventing the spread of infection, stating that individuals should not wear facemasks unless they are sick or caring for someone who is sick due to the national mask shortage [13]. However, new information confirms that asymptomatic COVID-19 patients can transmit the virus through direct contact with others and contamination of their environment [14–16]. In light of this information concerning asymptomatic transmission, more emphasis has been placed on wearing masks and physically distancing from others as a public health strategy.

Social distancing and other health behaviors are especially important for individuals with pre-existing conditions that put them at risk for complications due to COVID-19. Increased age, diabetes, hypertension, and other comorbidities are associated with increased ICU admissions, ventilator care, and deaths [17–19]. Additionally, cardiopulmonary conditions such as cardiovascular disease and chronic obstructive pulmonary disease are strong predictors of severe illness due to the virus [20].

Because COVID-19 is a novel virus, there is limited research related to predictors of adherence to important infection-mitigating behaviors related to COVID-19. Much of the existing COVID-19 research focuses on the epidemiology, mechanisms of transmission, development of therapeutic agents and vaccines, and risk factors. Considering this, existing literature on behaviors related to influenza are used in order to establish context for some of the variables of interest in this study. Existing literature suggests that gender differences exist concerning adherence to public health recommendations. Past research has shown that women adhere more to preventative health behaviors such as hand washing to reduce transmission of influenza than men do [21]. During the H1N1 crisis in 2009, a study found that women displayed higher perceived risk and demonstrated more precautionary behaviors related to H1N1 than men did [22]. These studies suggest that women tend to show a greater level of concern about health risks and adhere to more health recommendations in order to prevent illness related to viral infections. Considering this, we expect that women will adhere more to pandemic-related health guidelines than men.

The relationship between community size and adherence to pandemic-specific health behaviors is another variable of interest we examine in this study. In China, rural residents were found to adhere to recommended health behaviors like mask wearing, staying home, avoiding crowds, and frequent hand washing less frequently than those in urban communities [23]. Rural participants also report lower intention to adhere to these health recommendations, display a more pessimistic attitude about the effectiveness of complying to preventative health measures, and tend to be less knowledgeable about COVID-19 [24–26]. Given this information about the differences in health behaviors and attitudes between rural and urban residents, we expect that participants living in rural communities will adhere less to recommended health behaviors intended to prevent the spread of COVID-19.

Research on perceptions related to COVID-19 suggests that Americans have high confidence in the CDC as source of information [27]. However, other research implies that trust of the scientific community is negatively impacted when individuals are faced with information that challenges their personal political values [28]. Nisbet and colleagues found that individuals with low policy liberalism, or politically conservative viewpoints, tend to be more resistant to persuasion and react negatively to ideologically dissonant information than those with moderate to high policy liberalism. The researchers pose that level of policy liberalism influences how individuals evaluate information from the scientific community and that reading information that conflicts with personal ideology results in distrust of science. Individuals with low policy liberalism are generally less trusting of government medical experts while individuals with high policy liberalism are generally more receptive to information from the scientific community and show more concern related to the current pandemic [29, 30]. The political polarization of science is especially relevant during the current pandemic because adherence to recommended health guidelines will depend on trust of the scientific community. Given this information, we expect that participants with low policy liberalism will be the least trusting of organizations like the CDC and WHO. To our current knowledge, preexisting literature has only examined various platforms for news consumption, such as television, social media, or newspapers, as predictors of health behaviors [21, 31, 32]. This study is unique because both policy liberalism and partisanship of news sources will be examined as influences on COVID-19 related health behaviors.

The goal of this study is to identify what factors influence adherence to infection-mitigating health measures, perceived risk of personal health consequences due to COVID-19, and trust of the scientific community. We examine the effect of gender, community size, degree of policy liberalism, news sources, and attitudes about the pandemic on participants’ health behaviors. Considering the existing literature on patterns of adherence to recommended health behaviors, we expect that males with low policy liberalism who consume conservative news and are residents of small communities will have the lowest adherence to preventative health behaviors related to COVID-19. Additionally, we predict that the presence of pre-existing health conditions will be a motivating factor for adherence to preventative health behaviors. Through this research, we hope to expand the current understanding of COVID-19 and factors that influence individuals’ health behavior choices related to the pandemic.

## Method

The sample used in this study was restricted to United States citizens 18 years of age or older. All participants included in the study agreed to a statement of consent before answering the survey. No identifying information was collected so anonymity was protected. Participants were not offered any compensation or incentives for participation. This study was reviewed and approved by the Austin College Institutional Review Board (IRB # 2020–05) and adhered to APA ethical guidelines.

Data was collected via an online survey format using Microsoft Forms. Participants were recruited through Facebook, Twitter, and email. A snowball sampling method was used in order to achieve the largest possible sample within the timeframe of June 30, 2020 to July 22, 2020. A substantial effort was made to recruit a diverse sample by reaching out to ethnic minority groups and conservative communities on Facebook. The final sample size was 951 participants (M_age_ = 44.85, SD_age_ = 16.04, range = 18 to 83 years; 78.6% female; 86% White; Mdn_education level_ = Bachelor’s degree; Mdn_community size_ = City) (see Table 1). The survey consisted of three sections with 17 items total not including the statement of consent. The first section measured demographic information; age, gender, ethnicity, race, highest level of education, current employment status, and community size (see S1 Appendix for all measures). Questions on ethnicity and race were taken from the United States Census Bureau American Community Survey [33]. Categories for community size were based on criteria from the United States Census Bureau geography guide [34].

**Table 1:** Characteristics of participants in the sample (N = 951).

Sample Demographics	Frequency	Percentage
Age		
≤ 20	85	8.7
21–30	132	13.4
31–40	136	13.7
41–50	210	21.6
51–60	235	24.3
61–70	95	9.5
>70	57	5.7
Gender		
Male	202	20.7
Female	743	76.2
Racial Background		
White	838	85.9
Black	20	2.1
Asian	48	4.9
Hispanic	72	7.4
Native American/Pacific Islander	10	1.0
Multiracial	7	0.8
Education		
High School/GED	164	16.8
Bachelor’s Degree	364	37.3
Master’s Degree	287	29.4
PhD or equivalent	110	11.3
Trade School	18	1.8
Employment Status		
Retired	145	14.9
Unemployed	111	11.4
Employed Part-Time	127	13.0
Employed Full-Time	541	55.5
Community Population		
Town (1k-10k)	127	13.0
Large Town (10k – 100k)	217	22.3
City (100k-300k)	153	15.7
Large City (300k-1M)	217	22.3
Metropolitan Area (1M-3M)	171	17.5
High Density Area (>3M)	56	5.7

A 5-point Likert scale was used to measure attitudes on the importance of following protective measures and perceived reliability of information published by the CDC and WHO related to COVID-19 (e.g., “I can trust information about COVID-19 published by the CDC”) (Cronbach’s α = .85). Participants were also asked to indicate their perceived threat to personal health due to COVID-19 by selecting “*Very serious*”, “*Somewhat serious*”, or “*Not serious*”. The items measuring health information included any pre-existing health conditions, positive COVID-19 tests, degree of mental health impact, and compliance to both standard and pandemic-related hygiene recommendations. Participants were asked to indicate any health conditions that applied to them that put them at risk for complications due to COVID-19 (e.g., asthma, obesity, chronic lung disease). The number of health conditions reported by each participant was used in analyses.

For the purposes of this study, pandemic-related health behaviors (e.g., “I wear a cloth covering over my nose and mouth when I go out in public even if I do not feel sick”) are designated as recently implemented infection-mitigating behaviors specific to COVID-19 that are not part of traditional daily hygiene. General health behaviors (e.g., “I wash my hands for at least 20 seconds after using the toilet”) are designated as the standard health behaviors practiced daily to prevent the spread of ordinary pathogens. Compliance to pandemic-related hygiene recommendations was measured using a 5-point Likert scale consisting of three items (Cronbach’s α = .72), and compliance to general health behaviors was measured using a 5-point Likert scale consisting of five items (Cronbach’s α = .76). Participants were asked to indicate the frequency with which they engage in each health measure ranging from 1 “*Never*” to 5 “*Always*”. Separate composite subscores for both categories were created by averaging each of the individual scores together.

We utilized a 5-point Likert scale with an additional “Prefer not to say” option to assess degree of policy liberalism in economic, foreign, and nativist domains (Cronbach’s α = .81). The five items used in this scale were based on questions from a political typology assessment [35]. Participants were asked to rate their level of agreement with different statements (e.g., “In foreign policy, the U.S. should follow its own national interests even when its allies disagree”) from 1 “*Strongly disagree*” to 5 “*Strongly agree*”. Individual scores from each item were averaged together to yield a score representing policy liberalism with higher scores indicating more liberal attitudes and lower scores indicating more conservative attitudes. We chose to use this method instead of having participants self-report their political attitudes in order to mitigate self-reporting bias [36]. Participants were asked to indicate which news sources they use to stay informed. Multiple responses were allowed. To assess overall policy liberalism of news sources, responses that included sources with high policy liberalism (e.g., Huffington Post) were scored as 3 and responses that included sources with low policy liberalism (e.g., Fox News) were scored as 1. Responses that included only politically neutral sources were scored as 2. The Allsides media bias chart and University of Michigan Library research guide were used as references for classifying the overall policy liberalism of news sources [37, 38]. A blank was also provided for participants to add any sources that were not present on the list. An independent score representing the total number of conservative sources reported was used in analyses.

### Statistical analysis

All tests were conducted using SPSS version 26.0 [39]. Statistical analyses included Pearson’s correlation coefficient and standard multiple regression for continuous and dichotomous variables. Missing data was accounted for using pairwise deletion in order to minimize loss. The PROCESS modeling tool version 3.5 by Andrew Hayes [40] was used to analyze interactions between predictor variables. Cronbach’s α was used to measure internal reliability of items for pandemic-related health behaviors, general health behaviors, attitudes, and policy liberalism.

## Results

Bivariate correlations were estimated between variables of interest. All means, standard deviations, and inter-correlations are presented in Table 2. Participants who were female, had high policy liberalism, engaged in more general health behaviors, belonged to a larger community, had higher perceived risk, and consumed fewer conservative news sources reported higher compliance to pandemic-related health behaviors. The same variables also correlated with higher trust of the WHO. The strongest correlations between trust of the WHO and CDC were with level of policy liberalism and the number of conservative news sources utilized by participants.

**Table 2:** Bivariate correlations for the sample (range 895–940).

	1	2	3	4	5	6	7	8	9	10	11	12
1. COVID-19 Health Behaviors	-	.375**	.313**	-.078*	.072*	.140**	.155**	.103*	.325**	.029	.398**	-.378**
2. Level of Trust in the WHO		-	.730**	-.085*	-.124**	.109*	.172**	.118**	.071*	-.044	.509**	-.475**
3. Level of Trust in the CDC			-	-.063	-.084*	.033	.119**	.013	.081*	-.026	.317**	-.312**
4. Perceived Threat of COVID-19				-	-.025	.036	-.059	-.068*	-.038	-.049	-.104*	.035
5. Age					-	-.092*	.337**	-.003	.093*	.324**	-.036	.059
6. Gender						-	.028	.008	.254**	-.048	.122**	-.112*
7. Education Level							-	.148**	.028	.000	.239**	-.105*
8. Community Size								-	.026	-115**	.168**	-.115*
9. General Health Behaviors									-	.039	.058	-.033
10. # Preexisting Health Conditions										-	.029	.039
11. Policy Liberalism											-	-.539**
12. # Conservative News Sources												-

Note:

*p < .05,

**p < .001, Bold Correlations = Medium to High Effect Size; Gender (Male = 1, Female = 2).

**Fig 1:**
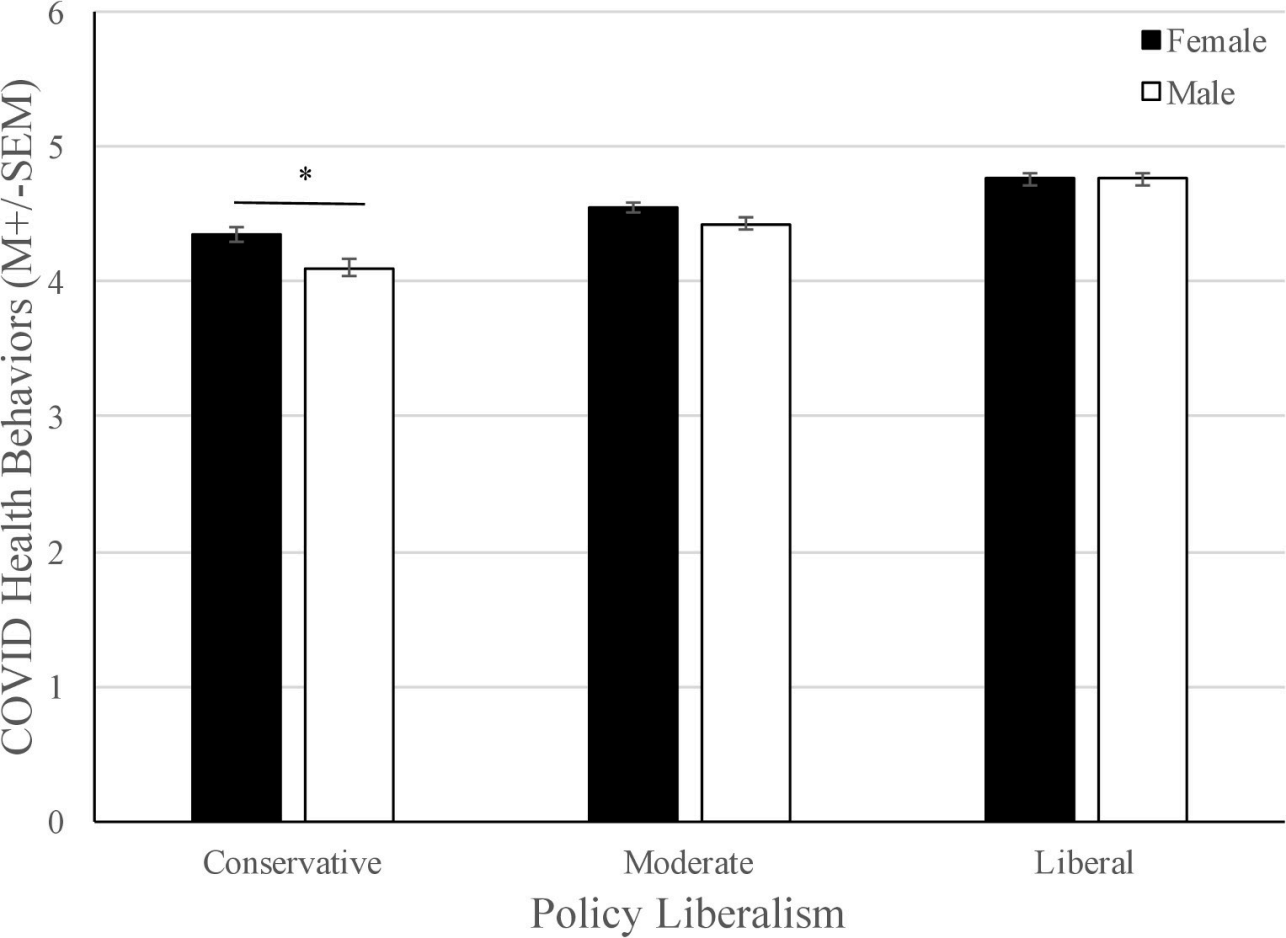
The interaction between policy liberalism and gender on adherence to COVID-19 specific health behaviors. Males and females high in policy liberalism did not differ in their adherence to COVID-19 specific health behaviors; however, males who have a conservative policy stance were much less likely to adhere to COVID-19 specific health behaviors, *F* (1,940) = 8.18, *p* = .004.

To determine which variables best predicted the adherence to pandemic-related health behaviors and trust of the scientific community, multiple linear regression analyses were conducted. The independent variables included in the analyses were gender, education level, age, community size, general health behaviors, number of health conditions, number of conservative sources used, level of policy liberalism, and the product term for gender-policy liberalism. Predictor variables for the regression models were chosen on the basis of theoretical relevance as these variables are central to our hypotheses. The regression models for pandemic-related health behaviors and trust of the WHO included a gender-policy liberalism product term because policy liberalism was found to moderate the relationship between gender and each of these outcome variables. *p* < .05 was considered significant. Multicollinearity was ruled out with all VIF < 10 and Tolerance > 0.10. See Table 3 for all standardized estimates and S1–S4 Tables for unstandardized coefficients, associated confidence intervals, and *p*-values. The overall model was found to be significant at predicting adherence to pandemic-specific health behaviors, adjusted *R*^*2*^ = 0.29, *F*(9, 885) = 41.99, *p* < .001. The best predictor variables in this model were gender, *β* = -.16, *t*(885) = -2.09, *p* = .04, policy liberalism, *β* = .37, *t*(885) = 5.95, *p* = < .001, number of conservative sources, *β* = -.23, *t*(885) = -6.72, *p* = < .001, and general health behaviors, *β* = .29, *t*(885) = 9.89, *p* = < .001. Since the interaction term between gender and policy liberalism was significant, *β* = .22, *t*(885) = 2.45, *p* = .01, PROCESS macro was used to probe the moderating effect at three levels of policy liberalism: one standard deviation above the mean, at the mean, and one standard deviation below the mean. Policy liberalism was mean centered prior to analysis. The results of this model showed that males who fell one standard deviation above the mean of policy liberalism had an overall lower average pandemic-related health behavior score than women falling one standard deviation above the mean (see Fig 1).

**Table 3:** Standardized estimates from regression analysis for pandemic-specific health behaviors, perceived risk, and trust of the scientific community.

	COVID-19 Health Behaviors	Trust of WHO	Trust of CDC	Perceived Risk
	*β*	*β*	*β*	*β*
Gender	-0.16*	-0.18*	-0.05	0.06
Age	0.05	-0.14***	-0.11**	0.01
Education Level	0.04	0.11***	0.10**	-0.03
Community Size	0.02	0.02	-0.06	-0.05
Number of Health Conditions	0.02	0.03	0.01	-0.06
General Health Behaviors	0.29***	0.05	0.08*	-0.04
Policy Liberalism	0.37***	0.45***	0.19***	0.11**
Number of Conservative News Sources	-0.23***	-0.27***	-0.20***	-0.03
Gender-Policy Liberalism	0.22*	0.23**	-	-
R^2^	0.30	0.34	0.15	0.02

Note:

*p < .05,

**p < .01,

***p < .001.

**Fig 2:**
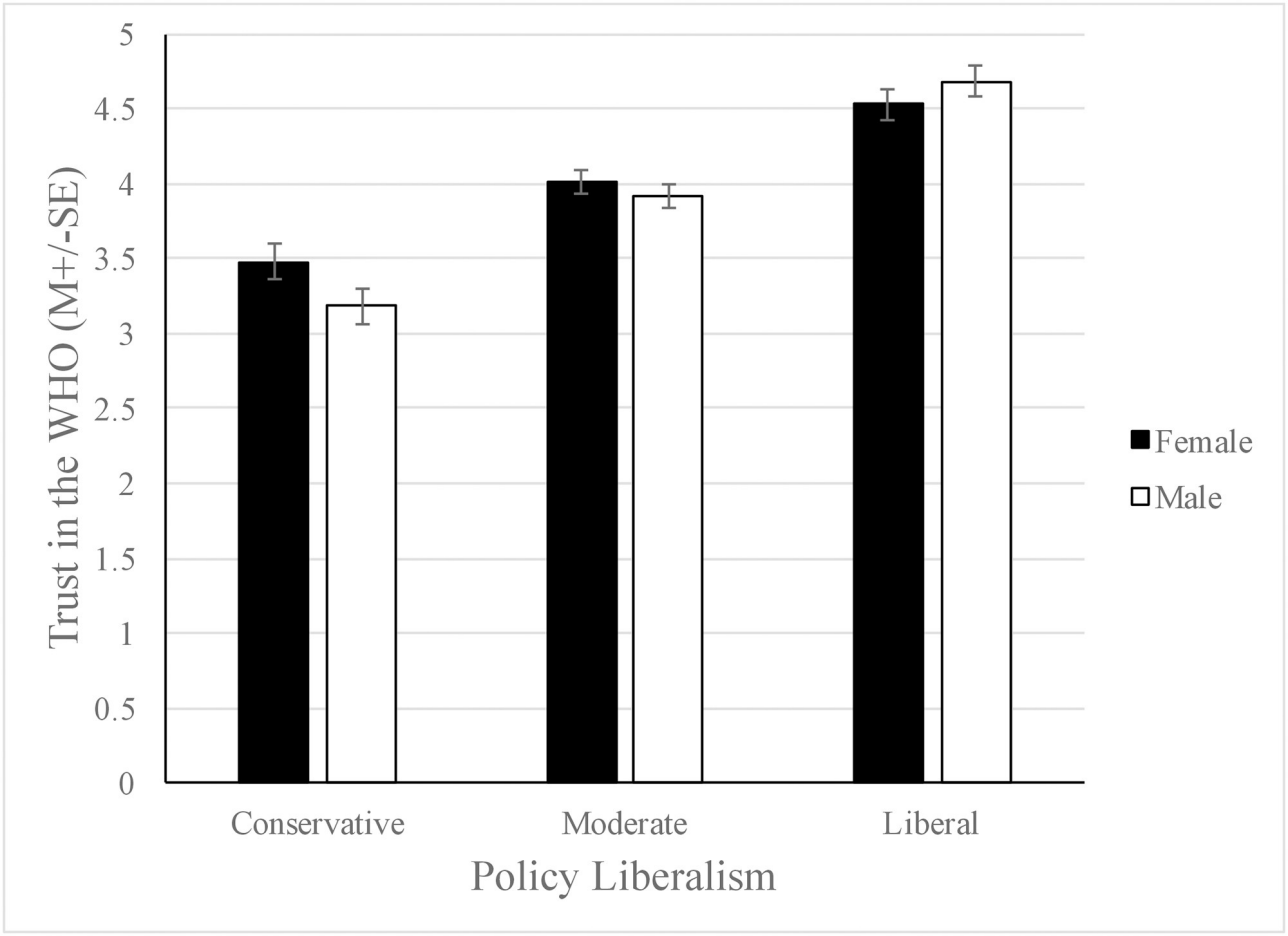
The interaction between policy liberalism and gender on trust of the WHO. Males and females scoring high in policy liberalism did not differ in their adherence trust of the WHO; however, males who hold a conservative stance were much less likely to trust the WHO, *F* (1,940) = 10.59, *p* = .001.

The model examining trust of the WHO using the same predictor variables was found to be significant, adjusted *R*^*2*^ = .34, *F*(9, 885) = 51.36, *p* < .001. The best predictor variables in this model were gender, *β* = -.18, *t*(885) = -2.32, *p* = .02, education level, *β* = .11, *t*(885) = 3.50, *p <* .001, age, *β* = -.14, *t*(885) = -4.58, *p* < .001, number of conservative sources consumed, *β* = -.27, *t*(885) = -8.31, *p* < .001, and policy liberalism, *β* = .45, *t*(885) = 7.60, *p* < .001. Since the interaction term between gender and policy liberalism was significant, *β* = .23, *t*(885) = 2.65, *p* = .008, the PROCESS macro was used to probe this interaction at three levels of policy liberalism. Policy liberalism was mean centered prior to analysis. Like the moderating effect of policy liberalism on gender as a predictor of compliance to pandemic-related health behaviors, conservative males demonstrated less trust of the WHO than did conservative females, while liberal participants of both genders scored similarly (see Fig 2). The model examining the trust of the CDC was also found to be significant, adjusted *R*^*2*^ = .14, *F*(8,886) = 19.34, *p* < .001, with the best predictor variables being age, *β* = -.11, *t*(886) = -1.42, *p* = .001, education level, *β* = .10, *t*(886) = 2.82, *p* = .005, general health behaviors, *β* = .08, *t*(886) = 2.58, *p* = .01, number of conservative sources consumed, *β* = -.20, *t*(886) = -5.45, *p* < .001, and level of policy liberalism, *β* = .19, *t*(886) = 5.02, *p* < .001. Although the effect size was small, the regression model used to examine perceived risk of COVID-19 was still significant, adjusted *R*^*2*^ = .01, *F*(8, 886) = 2.49, *p* = .011. The only significant predictor variable in this model was level of policy liberalism, *β* = .11, *t*(886) = 2.62, *p* = .009.

## Discussion

Our central findings reveal that degree of policy liberalism and use of conservative news sources by participants strongly predicted compliance to pandemic-related health behaviors, such as mask-wearing and social distancing, and trust in scientific organizations like the CDC and WHO. Contrary to our hypothesis, we did not find that number of health conditions predicted adherence to pandemic-related health measures. In addition, we found that policy liberalism moderated the relationship between gender and compliance to pandemic-related health behaviors. For liberal participants, there was no relationship between gender and policy liberalism but for conservative participants, males scored lower than females for pandemic-related health behaviors.

Our findings regarding gender differences are consistent with research confirming that men have a lower perceived risk of negative health impacts due to COVID-19 [41]. Despite these gender differences related to perceived risk, men have an immunological disadvantage when it comes to susceptibility to infectious diseases [42] and are disproportionately affected by adverse outcomes related to COVID-19 [43, 44]. We suspect that this paradox of men engaging in fewer health-seeking behaviors despite being unequally affected by negative health outcomes can be explained by cultural norms of “masculinity” that generally discourage men from seeking out needed health care services [45]. This norm of masculinity is especially present within the conservative subculture [46], which could further explain the moderating effects of policy liberalism on gender as a predictor variable of conformity to pandemic-related health practices.

Contrary to our hypothesis that the presence of pre-existing health conditions would predict compliance to pandemic-related health behaviors, we found no evidence to support this despite the concrete evidence that pre-existing conditions increase the risk of complications and mortality due to COVID-19 [47, 48]. Our findings could be explained by previous research indicating that individuals with pre-existing conditions underestimate their risk of mortality due to COVID-19 [49]. This has worrying implications that individuals with health conditions will experience disproportionate health consequences due to underestimation of risk.

Previous literature has shown that rural residents in the United States face significant barriers in obtaining preventative health care services like vaccinations due to the high number of uninsured individuals and geographic limitations leading to underutilization of services [50, 51]. Rural residents are typically older and have higher rates of co-morbidities and pre-existing health conditions, meaning that residents of rural communities are more susceptible to complications and mortality due to COVID-19 [52]. Literature investigating differences in preventative health behaviors related to COVID-19 between urban and rural residents in China found evidence that rural residents engaged in fewer pandemic-related health behaviors than urban residents [22, 25]. Evidence was also found that rural residents in the United States were less likely to stay home during the early months of the pandemic [53]. Considering this, we expected that participants from smaller communities would engage in fewer pandemic-related health behaviors. While we did not find that community size alone was a significant predictor of compliance, trends in the data revealed that participants who lived in small communities and consumed more conservative sources had the least trust of the WHO and the least conformity to pandemic-related health behaviors. We speculate that because prevalence of COVID-19 is greatest in areas with high population density, residents of smaller communities may have less incentive to adhere to CDC and WHO guidelines. In addition, we found that all participants, but especially those who live in small communities and consumed conservative news, had more trust in the CDC than the WHO. This could be attributed to the strong presence of cultural nationalism in the United States, which generally discourages trust in other nations and foreign organizations such as the WHO.

Other trends in the data revealed that participants younger in age with more education had more trust of the WHO. While neither age nor education level were significant predictors of compliance to pandemic-related health behaviors, both variables were significant predictors of trust in the scientific community. This could be explained by previous findings suggesting that education positively relates to trust in science [54]. We speculate that educated individuals may be better informed on current policy and public health issues and value information published by scientific institutions more than less educated individuals. Our findings that younger participants had more trust in the scientific community could be explained by the differences in news sources consumed by each age group. We did not find evidence that consumption of conservative news differed according to age, but younger participants were more likely to use social media as a source of information. Previous research has found evidence that individuals who utilize social media as a news source are more likely to trust the scientific community due to the likelihood that they will come across scientific news and be connected to current information [55].

Our findings suggest that while increased perceived risk was associated with higher conformity to pandemic-related health behaviors, perceived risk alone was not a significant predictor of adherence to these recommended health practices. This is inconsistent with prior research showing that risk perception is a very strong predictor of compliance to pandemic-related health behaviors [56]. However, our findings indicate that perceived risk was substantially moderated by policy liberalism, which could provide an explanation as to why perceived risk alone was not a significant predictor of these behaviors.

The role of policy liberalism and consumption of conservative news sources as predictors for our outcome variables was perhaps the most critical of our findings. Recent literature suggests that policy liberalism plays a considerable role in influencing perceived risk and infection-mitigating behavior changes related to the pandemic [57, 58]. In line with this research, we found evidence that policy liberalism was a strong and common predictor for both compliance to pandemic-related health practices and trust of the scientific community. There is evidence that conservatives have lower trust in science than liberals [59] and have less perceived risk about threatening phenomena such as global warming [60]. A study conducted in 2013 investigating the role of trust in the scientific community in the relationship between conservative media and beliefs related to global warming found that a greater use of conservative news sources was associated with high skepticism of global warming and low trust in the scientific community [61]. This is consistent with our findings that consuming more conservative media is associated with less trust of the scientific community.

Because we used a non-probability sampling method, we are unable to determine sampling error or make conclusions about the United States population as a whole. The correlational study design limits our ability to establish causes of pandemic-related behavior changes and perceived risk. Likewise, self-reported survey data is subject to bias, especially regarding politically-focused questions. In addition, our survey is not nationally representative, as only 16% of our sample was comprised of ethnic minorities. Another limitation of this study is that our survey did not include use of alcohol-based sanitizers as a preventative measure. Also, we did not utilize an established multi-item risk perception index. Only one survey item assessed degree of perceived risk to personal health so our measures are not quite as comprehensive as other recent publications. Only 2% of the variance in our regression model predicting perceived risk could be accounted for, so the majority of the variance has yet to be explained. Prior research has indicated that both knowledge and first-hand experience with COVID-19 can influence risk perception [41, 57], which are variables we did not measure in this study. Variables not assessed could contribute to unexplained variance in the model. Finally, this study was conducted from June to July of 2020, so our findings represent the attitudes and health behaviors of participants in the early stages of the pandemic, which may vary from the current attitudes and practices as confirmed cases in the United States are nearing 29 million and deaths have exceeded 500,000 [62]. We encourage future research to consider these limitations and expand upon our findings.

## Conclusions

Our research is unique because we found evidence that conservative news sources contribute to low conformity to recommended health practices and high distrust of the scientific community as it relates to the pandemic. The moderating effect on the relationship between gender and adherence to pandemic-related health behaviors was a key finding of the study. Another significant finding of the study was the role of news sources and policy liberalism as predictors of conformity to pandemic-related health guidelines and trust in the scientific community. We expect that these behaviors can be explained by conservative news sources minimizing the severity of this health crisis and dismissing the importance of adhering to infection mitigating protocols while disparaging the scientific organizations that establish these guidelines. Our research contributes to the growing body of evidence suggesting that policy liberalism and the consumption of conservative news play a larger role in individuals’ motivations to adhere to recommended health practices than established risk factors. These conclusions point to concerning ramifications on the health and wellbeing for Americans as the pandemic progresses.

## Supporting information

S1 Table Regression model predicting compliance to pandemic-related health behaviors. (DOCX)

S2 Table Regression model predicting trust of the WHO. (DOCX)

S3 Table Regression model predicting trust of the CDC. (DOCX)

S4 Table Regression model predicting perceived risk of COVID-19. (DOCX)

S1 Appendix Study measures. (DOCX)
