# Supplementary material for: Policy liberalism and source of news predict pandemic-related health behaviors and trust in the scientific community
Source: PLoS One. 2021 Jun 17;16(6):e0252670. doi: 10.1371/journal.pone.0252670 (PMC8211217; doi:10.1371/journal.pone.0252670)
Supplement: S1 Table — (DOCX) [file pone.0252670.s001.docx]

**S1 Table.** Regression model predicting compliance to pandemic-related health behaviors.

|  | COVID-19 Health Behaviors | | | |
| --- | --- | --- | --- | --- |
|  | *B* | 95% CI | *SE* | *β* |
| Gender | -0.25 | [-0.48,-0.01] | 0.12 | **-0.16*** |
| Age | 0.002 | [-0.001,0.004] | 0.001 | 0.05 |
| Education Level | 0.02 | [-0.01,0.05] | 0.02 | 0.04 |
| Community Size | 0.01 | [-0.01,0.03] | 0.01 | 0.02 |
| Number of Health Conditions | 0.02 | [-0.02,0.06] | 0.02 | 0.02 |
| General Health Behaviors | 0.28 | [0.22,0.33] | 0.03 | **0.29***** |
| Policy Liberalism | 0.24 | [-0.32,-0.16] | 0.04 | **0.37***** |
| Number of Conservative News Sources | -0.26 | [-0.34,-0.19] | 0.04 | **-0.23***** |
| Gender-Policy Liberalism | 0.11 | [0.02,0.19] | 0.04 | **0.22*** |
| *R*^2^ | **0.30** | | | |

Note: **p* < .05, ***p* < .01, ****p* < .001, Gender (Male = 1, Female = 2).
