# Supplementary material for: Policy liberalism and source of news predict pandemic-related health behaviors and trust in the scientific community
Source: PLoS One. 2021 Jun 17;16(6):e0252670. doi: 10.1371/journal.pone.0252670 (PMC8211217; doi:10.1371/journal.pone.0252670)
Supplement: S2 Table — (DOCX) [file pone.0252670.s002.docx]

**S2 Table**. Regression model predicting trust of the WHO.

|  | Trust of WHO | | | |
| --- | --- | --- | --- | --- |
|  | *B* | 95% CI | *SE* | *β* |
| Gender | -0.51 | [-0.95,-0.08] | 0.22 | **-0.18*** |
| Age | -0.01 | [-0.02,-0.01] | 0.002 | **-0.14***** |
| Education Level | 0.10 | [0.05,0.16] | 0.03 | **0.12***** |
| Community Size | 0.01 | [-0.03,0.06] | 0.02 | 0.02 |
| Number of Health Conditions | 0.03 | [-0.04,0.11] | 0.04 | 0.03 |
| General Health Behaviors | 0.09 | [-0.01,0.19] | 0.05 | 0.05 |
| Policy Liberalism | 0.57 | [-0.72,-0.42] | 0.07 | **0.45***** |
| Number of Conservative News Sources | -0.61 | [-0.75,-0.46] | 0.07 | **-0.27***** |
| Gender-Policy Liberalism | 0.21 | [0.06,0.37] | 0.08 | **0.23**** |
| *R*^2^ | **0.34** | | | |

Note: **p* < .05, ***p* < .01, ****p* < .001, Gender (Male = 1, Female = 2).
