# Supplementary material for: Policy liberalism and source of news predict pandemic-related health behaviors and trust in the scientific community
Source: PLoS One. 2021 Jun 17;16(6):e0252670. doi: 10.1371/journal.pone.0252670 (PMC8211217; doi:10.1371/journal.pone.0252670)
Supplement: S3 Table — (DOCX) [file pone.0252670.s003.docx]

**S3 Table.** Regression model predicting trust of the CDC.

|  | Trust of CDC | | | |
| --- | --- | --- | --- | --- |
|  | *B* | 95% CI | *SE* | *β* |
| Gender | -0.12 | [-0.28,0.05] | 0.08 | -0.05 |
| Age | -0.01 | [-0.01,-0.003] | 0.002 | **-0.11**** |
| Education Level | 0.09 | [0.03,0.14] | 0.03 | **0.10**** |
| Community Size | -0.04 | [-0.09,0.003] | 0.02 | -0.06 |
| Number of Health Conditions | 0.01 | [-0.06,0.09] | 0.04 | 0.01 |
| General Health Behaviors | 0.14 | [0.03,0.24] | 0.05 | **0.08*** |
| Policy Liberalism | 0.22 | [-0.30,-0.13] | 0.04 | **0.19***** |
| Number of Conservative News Sources | -0.40 | [-0.55,-0.26] | 0.07 | **-0.20***** |
| *R*^2^ | **0.15** | | | |

Note: **p* < .05, ***p* < .01, ****p* < .001, Gender (Male = 1, Female = 2).
