# Supplementary material for: Policy liberalism and source of news predict pandemic-related health behaviors and trust in the scientific community
Source: PLoS One. 2021 Jun 17;16(6):e0252670. doi: 10.1371/journal.pone.0252670 (PMC8211217; doi:10.1371/journal.pone.0252670)
Supplement: S4 Table — (DOCX) [file pone.0252670.s004.docx]

**S4 Table.** Regression model predicting perceived risk of COVID-19.

|  | Perceived Risk | | | |
| --- | --- | --- | --- | --- |
|  | *B* | 95% CI | *SE* | *β* |
| Gender | 0.10 | [-0.02,0.21] | 0.06 | 0.06 |
| Age | 0.00 | [-0.003,0.004] | 0.002 | 0.01 |
| Education Level | -0.02 | [-0.06.-0.02] | 0.02 | -0.03 |
| Community Size | -0.03 | [-0.06,0.006] | 0.02 | -0.05 |
| Number of Health Conditions | -0.05 | [-0.10,0.01] | 0.03 | -0.06 |
| General Health Behaviors | -0.05 | [-0.12,0.03] | 0.04 | -0.04 |
| Policy Liberalism | 0.08 | [0.02,0.14] | 0.03 | **0.11**** |
| Number of Conservative News Sources | -0.03 | [-0.14,0.07] | 0.05 | -0.03 |
| *R*^2^ | **0.02** | | | |

Note: **p* < .05, ***p* < .01, ****p* < .001, Gender (Male = 1, Female = 2).
