# Supplementary material for: Policy liberalism and source of news predict pandemic-related health behaviors and trust in the scientific community
Source: PLoS One. 2021 Jun 17;16(6):e0252670. doi: 10.1371/journal.pone.0252670 (PMC8211217; doi:10.1371/journal.pone.0252670)
Supplement: S1 Appendix — (DOCX) [file pone.0252670.s005.docx]

**S1 Appendix**. Study measures.

1. Please input the date that you are taking this survey

Demographic Information (US Census Bureau, 2020)

2. What is your age?

3. Please indicate your gender

• Male

• Female

• Other (free response)

• Prefer not to say

4. Are you of Hispanic, Latino, or Spanish origin?

• Yes

• No

• Prefer not to say

5. Please specify the race you identify with the most

• White

• Black or African American

• Asian

• American Indian or Alaska Native

• Native Hawaiian or Other Pacific Islander

• Prefer not to say

• Other (free response)

6. What is your highest level of education?

• Middle School only

• Some High School

• High School or GED

• Bachelor’s Degree

• Master’s Degree

• Ph. D. or higher

• Trade School

• Prefer not to say

7. What is your current employment status?

• Employed full-time

• Employed part-time

• Unemployed or Seeking Employment

• Retired

• Prefer not to say

8. What is the approximate size of your community?

• Town (1,000 to 10,000 people)

• Large town (10,000 to 100,000 people)

• City (100,000 to 300,000 people)

• Large city (300,000 to 1 million people)

• Metropolis (1 million to 3 million people)

• Very high density (3 million or more)

Attitudes and Trust of the Scientific Community

9. Please indicate how strongly you feel about the following statements

• Strongly Disagree

• Somewhat Disagree

• Neutral

• Somewhat Agree

• Strongly Agree

1. I can trust information about COVID-19 published by the CDC

2. I can trust information about COVID-19 published by the WHO

3. It is important to practice social distancing (i.e. maintaining 6-foot distance between myself and others)

4. It is important to wear a mask or a covering on my nose and mouth when I am in a public space

5. It is important to routinely clean surfaces with disinfectants to reduce the spread of infection

Perceived Risk Assessment

10. How much of a health threat do you perceive COVID-19 to be to your health?

• Very serious –skip 12

• Somewhat serious –skip 11 and 12

• Not serious –skip 11

11. Please indicate why you perceive COVID-19 to be a serious health threat

• I am immunocompromised or at risk for serious illness as a result of COVID-19

• I know someone who is immunocompromised or at risk for serious illness as a result of COVID-19

• I want to avoid getting ill

• Other (free response)

12. Please indicate why you do not perceive COVID-19 to be a serious health threat

• The media is exaggerating the seriousness of the virus

• I am young and not at risk of experiencing complications from the virus

• The virus is no more dangerous than the flu

• I have a strong immune system and will not contract the virus

• Other (free response)

Measures of Health and Risk Factors

13. Please indicate which health conditions apply to you (Please select all that apply)

• Moderate to severe asthma

• Chronic kidney disease

• Chronic lung disease

• Diabetes

• Obesity

• Heart condition

• Immunocompromised

• Over the age of 65

• Liver disease

• Undergoing cancer treatment

• Living in a nursing home or assisted living facility

• Prefer not to say

• I have none of these conditions

14. Have you contracted and tested positive for COVID-19 this year?

• Yes

• No

14. Please indicate the degree to which the pandemic has negatively impacted your mental health

(Not at All) 1 2 3 4 5 (Severely)

Health Behaviors Assessment (CDC, July 2020)

15. How often do you comply with the following hygiene recommendations?

• Always

• Almost always

• Half of the time

• Rarely

• Never

1. I wash my hands for at least 20 seconds before, during, and after preparing food

2. I wash my hands for at least 20 seconds after using the toilet

3. I wash my hands for at least 20 seconds after blowing my nose, coughing, or sneezing

4. I wash my hands for at least 20 seconds when I enter my house after being outside

5. I disinfect appliances like my phone and surfaces like countertops daily

6. I cover my coughs and sneezes with a tissue or the inside of my elbow

7. I wear a cloth covering over my nose and mouth when I go out in public even if I do not feel sick

8. I put 6 feet of distance in between myself and others

9. I stay out of crowded places

News Sources

10. Please indicate the news sources you use to stay informed (Please select all that apply)

• ABC News

• CBS News

• CNN

• Fox News Channel

• Drudge Report

• Infowars

• MSNBC

• NBC News

• The New York Times

• Los Angeles Times

• One America News Network (OAN)

• USA Today

• Breitbart News Network

• The Washington Post

• The Wall Street Journal

• Vice News

• HuffPost

• NPR

• Time

• The Guardian

• Social Media (Facebook, etc.)

• Other (free response)

Political Leaning Assessment (Pew Research Center, 2020)

11. Which of the following statements comes closest to your political view?

- Strongly Disagree
- Somewhat Disagree
- Neutral
- Somewhat Agree
- Strongly Agree
- Prefer not to say

1. The economic system in the US is generally fair to most Americans

2. Most people who want to get ahead can make it if they are willing to work hard

3. In foreign policy, the US should follow its own national interests even when its allies disagree

4. The US should pay less attention to problems overseas and concentrate on problems here at home

5. Immigrants today are a burden on our country because they take jobs, housing, and healthcare
